# Nashville Monthly Record of Medical and Physical Science

**Published:** 1858-11

**Authors:** 


NASHVILLE MONTHLY RECORD OF MEDICAL & PHYSICAL SCIENCE.

We have been for some time in receipt of this new Medical Journal and regret our failure to notice it before, as its merits had attracted our particular attention.

We now most warmly commend it to the profession and welcome it to the list of our exchanges, with an earnest desire for its most eminent success.

The gentlemen who have charge of it, Drs. D. F. Wright and R. O. Currey, are admirably fitted for their position and will, we are sure, furnish a first-class journal—this, indeed, is no prediction, as it is already clearly demonstrated in the number before us.

It is published monthly at $2 per annum, at Nashville, Tenn.

